# Effect of Dispersion-Enhanced Sensitivity in a Two-Mode Optical Waveguide with an Asymmetric Diffraction Grating

**DOI:** 10.3390/s21165492

**Published:** 2021-08-15

**Authors:** Andrei Tsarev

**Affiliations:** 1 Laboratory of Optical Materials and Structures, Rzhanov Institute of Semiconductor Physics, SB RAS, 630090 Novosibirsk, Russia; tsarev@isp.nsc.ru or a.tsarev@g.nsu.ru; Tel.: +7-913-4810-578; 2 Department of Physics, Novosibirsk State University, 630090 Novosibirsk, Russia

**Keywords:** optical sensors, bimodal interaction, silicon wire, segmented grating, numerical modeling, finite difference time domain (FDTD) method

## Abstract

Analysis of trends in the development of silicon photonics shows the high efficiency regarding the creation of optical sensors. The concept of bimodal sensors, which suggests moving away from the usual paradigm based only on single-mode waveguides and using the inter-mode interaction of guided optical waves in a two-mode optical waveguide, is developed in the present paper. In this case, the interaction occurs in the presence of an asymmetric periodic perturbation of the refractive index above the waveguide surface. Such a system has unique dispersion properties that lead to the implementation of collinear Bragg diffraction with the mode number transformation, in which there is an extremely high dependence of the Bragg wavelength on the change in the refractive index of the environment. This is called the “effect of dispersion-enhanced sensitivity”. In this paper, it is shown by numerical calculation methods that the effect can be used to create optical sensors with the homogeneous sensitivity higher than 3000 nm/RIU, which is many times better than that of sensors in single-mode waveguide structures.

## 1. Introduction

Silicon photonics [1,2,3,4,5,6] are promising technologies with huge potential for the mass production of modern optical elements and functional systems based upon them. Optical sensors [7,8,9,10,11,12,13] are among the most popular components of information technologies systems that are manufactured, among other things, on the basis of silicon photonics and nanotechnologies. For example, over the past 10 years, 20% of all publications on sensors have been based on silicon photonics technologies, and 20% of publications on silicon photonics are devoted to optical sensors [13]. The main factors influencing the scale of optical sensor application include the sensitivity (S), intrinsic limit of detection (iLOD), and manufacturability. Sensor research is conducted in the most developed countries, and, in recent years, various types of sensors with very high technical parameters have been developed using various design solutions and the highly advanced technologies [7,8,9,10,11,12,13]. There is also a fundamental possibility of increasing optical sensor sensitivity by using two-mode optical waveguides in which bimodal interaction is implemented [14,15,16,17,18,19,20,21,22,23,24,25,26,27]. Bimodal interaction between the guided and leaky modes in a silicon waveguide with a side grating provides very high sensitivity [28]. In this paper, focus is placed on study of the dispersion properties of guided wave intermodal interaction in two-mode optical waveguides in silicon-on-insulator (SOI) structures with an asymmetric periodic grating on the top.

The study of guided wave propagation in optical waveguides in the presence of the periodic perturbation of their properties has been a classical problem of integral optics since the origin of the field. With the development of technology, the tasks solved have become more diverse, and the results obtained have gradually moved from fundamental findings to the field of practical use. Usually, the subject of research is the optical phenomena observed when an optical wave propagates along an optical waveguide (in our case, a silicon wire) in the presence of a diffraction grating as a structure with a periodically changing refractive index and/or a waveguide boundary [29,30]. The presence of a diffraction grating can lead to the coupling of various optical waves that propagate in such a structure, usually called grating-assisted couplers. The interaction is maximal when the Bragg synchronism condition is met for the interacting modes with an effective refractive index N_i_:

Abs(k_0_ − k_1_) = K p (1)

where k_i_ = 2πN_i_/λ_0_ is the wave number of the i-th mode, i = 1 or 2, N_i_ is the effective refractive index of modes (mode index), λ_0_ is the optical wavelength, K = 2 π/Λ is the diffraction grating wave number, Λ is the diffraction grating period, and p is the diffraction order. Here, we consider the interaction in the first order of diffraction (p = 1), which has the maximum efficiency of collinear intermodal interaction, as well as providing the operation in the telecommunications wavelength range.

The current state of research on this issue has been analyzed in detail in many reviews [7,8,9,10,11,12,13]. In particular, the optical properties of the waveguide mode depend on the refractive index of the surrounding medium n_c_, for example, a liquid, and its effective refractive index changes with a change in the wavelength, λ_0_, and the refractive index of the latter, N_i_ = N_i_(n_c_, λ_0_). According to Equation (1), when an optical wave propagates along a waveguide with a periodic grating, the optical wavelength λ_0_, at which the propagation is blocked (filtered), will also depend on the mode index N_i_, and, consequently, on the refractive index of the environment n_c_.

(N_0_ ± N_1_)⋅λ_0_= Λ (2)

This property is used to create optical sensors [31,32,33,34,35,36,37]. The homogeneous sensitivity S_n_ is described by the value of the change in the filtration wavelength with a change in the refractive index of the environment:

S_n_ = ∂λ/∂n_c_ (3)

Another important sensor parameter is the intrinsic limit of detection, which can be written as:

iLOD = δλ/S_n_ (4)

where δλ is the 3 dB wavelength bandwidth that determines the quality factor Q = λ/δλ of the sensor filter element, for example, a Bragg grating or a ring resonator.

To increase the sensitivity of a sensor based on a channel waveguide, an optical mode close to the cut-off is usually used, i.e., the waveguide dimensions are chosen such that the guided mode is still supported by the waveguide, but its effective refractive index is close to the refractive index of the environment. In this case, the decaying electromagnetic field components of the optical mode penetrate far into the surrounding space, which ensures the high sensitivity of such sensors. For a sensor in water, it is of the order of 70–100 nm/RIU and 200 nm/RIU for TE and TM polarizations, respectively [32]. This corresponds to the wavelength shift of 200 nm when the environment refractive index changes by 1 unit (RIU).

To further increase the sensitivity of a sensor, slot optical waveguides can be used, in which there is a narrow gap (about 100 nm) filled with the environment. Since the slot is filled with a medium with a low refractive index, a high wave field concentration is observed in this case, which increases the sensitivity of such a sensor element to S_n_ = 300 nm/RIU.

The diffraction grating can be constructed as a segmented periodic structure obtained by vertical etching. Subwavelength waveguide gratings (SWG) with a small period (Λ << N_i_/λ_0_) are effectively used for engineering the optical properties of waveguides, as well as for creating optical sensors. Their properties are correctly described by the continuous medium theory, which implies that a subwavelength segmented periodic structure is equivalent to a homogeneous medium with a refractive index equal to the average refractive index of the structure (for the case of TE polarization). If the segmented waveguide is surrounded by a medium whose properties are of interest, then the effective refractive index of the waveguide mode is very sensitive to the changes in the refractive index of the environment, which increases the sensor sensitivity (S_n_ = 400–500 nm/RIU). The use of inhomogeneous segment structures further increases the homogeneous sensitivity of a sensor (up to 617 nm/RIU) [33]. A further increase in sensitivity (S_n_ =1500 nm/RIU) has been observed in a photonic crystal-based resonator [34]. This was achieved by engineering the optical properties of a photonic crystal with the optimal parameters for its structure.

Although subwavelength waveguide arrays and slot waveguides significantly increase the sensitivity of sensors based on the use of a uniform waveguide, they have a significant disadvantage that is especially manifested when controlling the refractive index of liquid media. The fact is that high sensitivity of slot waveguides and subwavelength segment waveguides, as well as structures based on photonic crystals, is observed if the characteristic size of the etching groove is in the order of 100 nm. Consequently, there is the problem of incomplete filling with a liquid (due to viscosity and wetting) where narrow grooves form slot and segment waveguides, and that leads to a decrease in sensitivity [35] and an increase in the response time of optical sensors. In addition, for manufacturing, it is necessary to use sufficiently advanced and expensive technologies that provide precision for the vertical etching of small-sized elements (about 100 nm), and this fact does not contribute to the widespread use of such sensors.

In recent studies [36,37], to increase the sensitivity of optical sensors, it has been proposed to use the anomalous blocking effect, which is observed in a silicon waveguide that is tunneled with a segment periodic structure of a relatively large period (about 1.4 μm). This segment diffraction grating exhibits the properties of a leaky waveguide, the effective refractive index of which strongly depends on the refractive index of the surrounding medium. In such a structure, the diffraction grating carries a double load, where it forms the leaky waveguide and couples the fundamental mode of the silicon waveguide with the leaky mode of the segment structure. Sensors based on the anomalous blocking effect have a high sensitivity (S_n_ =420 nm/RIU) that is comparable to the sensitivity of sensors manufactured using alternative technologies based on slot and segment waveguides; however, due to the significantly larger characteristic size of the etching slots, they are more technologically advanced. What is especially important is that they do not have the problem of the incomplete filling of the slots with the surrounding liquid, which reduces the sensory properties of competitive technologies. A significant disadvantage of sensors based on the anomalous blocking effect is the relatively low intrinsic limit of detection, which is associated with big losses of the leaky wave of segment waveguide, and, consequently, with a wide filtration bandwidth.

Readouts of the information obtained from optical sensors are usually realized by narrow-band structures based on ring resonators [31,32,33], photonic crystals [34], and Mach–Zehnder interferometers [38], as well as Bragg grating-based structures [39,40,41].

## 2. Sensor Design

Recent publications, as well as the results of the author’s own numerical simulations, suggest a promising research direction which consists of studying the intermodal interaction in a two-mode optical waveguide with a periodically asymmetric diffraction coupling element, which can be the basis for optical sensors with a unique sensitivity (several µm/RIO range).

The starting point is the result of the study of a notch optical filter [42] constructed by a two-mode optical waveguide with an asymmetric grating-assisted coupling element. This consists of the silicon strip waveguide and two segmented diffraction gratings shifted by half a period and placed in the waveguide vicinity. Such an asymmetric diffraction grating provides an efficient transformation between the symmetric fundamental TE_0_ mode and the asymmetric TE_1_ mode at a wavelength that meets the condition of Bragg synchronism.

A similar type of collinear diffraction which implements the intermodal interaction between the fundamental and first asymmetric leaky outgoing modes in a waveguide with a grating manufactured on one side has recently been used to create optical sensors with an experimentally reached sensitivity of 5.08 µm/RIU [28]. The results of numerical modeling by the FDTD method for an optical sensor operating on the basis of interference of the fundamental and first modes in a subwavelength periodic waveguide may also be noted in this regard [26]. This has provided a significant sensitivity increase (up to 2.5 times) compared to the known types of sensors based on single-mode waveguides in similar structures; however, it should be noted that the maximum sensitivity of 1.3 µm/RIU is observed only in the long wavelength part of the optical spectrum (1665 nm). Furthermore, even in the telecommunication range (1550 nm), this sensor shows a relatively high sensitivity of 400 nm/RIU.

The diffraction grating formation by the precision deep etching at a submicron and strictly specified distance from the waveguide used in [42] is a complex technological problem, and this technology is not easily applied in the mass production of optical sensors.

**Figure 1:**
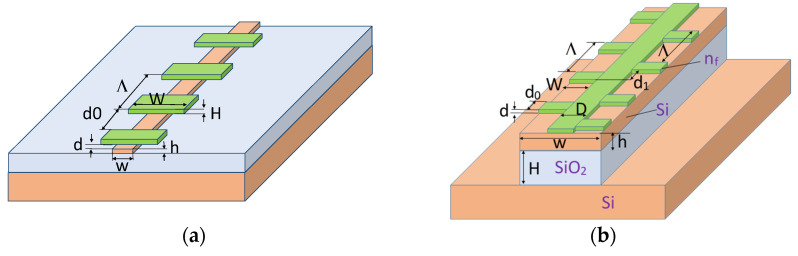
Grating coupling element based on a silicon optical waveguide in the SOI structure. (**a**) Waveguide structure with a symmetric grating for the TE_0_ - TE_0_ diffraction. (**b**) Waveguide structure with an asymmetric grating for the TE_0_-TE_1_ diffraction where n_f_ is refractive index of segmented grating (in our case polymer SU-8). The meaning of the structure parameters is evident from the figure and are the subject of the optimization for particular sensor design.

In this paper, an asymmetric diffraction grating is proposed for use, where the grating is made directly above the silicon waveguide location region (see Figure 1b) as a thin oxide or polymer (for example, SU-8) layer with a precisely controlled thickness that determines the intermodal interaction efficiency. Monitoring the oxide or the polymer film thickness is a more reproducible technology than observing the specified width of a narrow etching groove (about 200 nm), and the structure proper has unique dispersion properties that are suitable for increasing the sensitivity of optical sensors.

To study the intermodal interaction, the behaviors and interactions of guided modes (TE_0_ and TE_1_) propagating along a two-mode optical silicon wire waveguide in the presence of a segment diffraction grating (see Figure 1a,b) have been numerically modeled. For the analysis, the Rsoft numerical packages were used, which implement the beam propagating method (BPM), the finite element method (FEM), and the finite difference time domain method (FDTD) as provided by Synopsis [43]. All these algorithms have proven their accuracy and efficiency in solving similar problems [26,28,36,37,41].

The numerical three-dimensional analysis by the 3D FDTD method shows that the symmetric diffraction grating (see Figure 1a) with a period of 0.38 μm at a wavelength of about 1.53 μm provides an effective Bragg reflection of the fundamental TE_0_ mode that is observed in a silicon waveguide with thickness h = 0.25 μm and width w = 0.35 μm. Since this waveguide is close to the fundamental mode cutoff, it has a relatively high sensitivity (S_n_ = 190 nm/RIU) for changing the position of the Bragg synchronism wavelength to a perturbation of the refractive index of the surrounding medium, i.e., water in this case (n_c_ = 1.33).

As the silicon wire width increases (w > 0.49 μm), the waveguide starts to support the second mode; however, due to the different symmetry of the fundamental and first modes of the optical waveguide, the diffraction with a change in the mode number on the symmetric grating is impossible. Consequently, an asymmetric diffraction grating (see Figure 1b) is used, which is located on the top of the waveguide. In case of the diffraction grating with the period of 0.38 μm at the wavelength of 1.54 μm, one may observe the effective back diffraction from the TE_0_ mode to the TE_1_ mode. As the waveguide width w = 0.5 µm is close to the first mode cut-off point, this design provided a moderate sensitivity of S_n_ = 220 nm/RIU.

The sensitivity of a sensor based on the intermodal interaction on a diffraction grating can be quantified from Equation (2) [28] for the condition of phase Bragg synchronism. To do this, one may differentiate expression Equation (2) by taking into account the mode dispersion:

∂λ/∂n_c_ = Λ⋅(∂N_0_/∂n_c_ ± ∂N_1_/∂n_c_) + Λ⋅(∂N_0_/∂λ ± ∂N_1_/∂λ)⋅∂λ/∂n_c_ (5)

From this relationship, we obtain the following expression:

∂λ/∂n_c_ = Λ⋅(∂N_0_/∂n_c_ ± ∂N_1_/∂n_c_)/[1 − Λ (∂N_0_/∂λ ± ∂N_1_/∂λ)] (6)

One may substitute the determination for the group velocity N_ig_ = c⋅∂k/∂ω = N_i_ − λ_0_⋅∂N_i_/∂λ and Equation (2) and get the desired expression for the homogeneous sensitivity:

S_n_ = ∂λ/∂n_c_ = Λ (N_0_ ± N_1_)×(∂N_0_/∂n_c_ ± ∂N_1_/∂n_c_)/(N_0g_ ± N_1g_) (7)

Alternatively, this can be written in the following form:

S_n_ = ∂λ/∂n_c_ = λ_0_⋅(∂N_0_/∂n_c_ ± ∂N_1_/∂n_c_)/(N_0g_ ± N_1g_) (8)

where the sign is responsible for the propagation direction of the diffracted wave relative to the incident one, i.e., “+” denotes backward diffraction and “−” denotes forward diffraction. Furthermore, ω and c denote the circular frequency and speed of light in a vacuum, respectively.

Note that Equation (8) [28] was obtained by taking into account the optical waveguide dispersion properties. For example, if the dispersion is ignored, a wrong expression is obtained for the homogeneous sensitivity based only on the first term of Equation (6):

S_n_ = ∂λ/∂n_c_ = Λ⋅(∂N_0_/∂n_c_ ± ∂N_1_/∂n_c_) (9)

**Figure 2:**
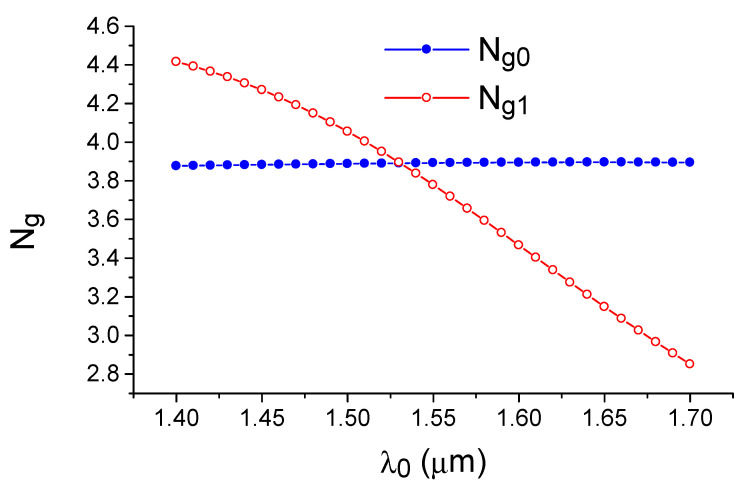
Group index dependence of the fundamental TE_0_ and first modes TE_1_ of a silicon waveguide on the optical wavelength. The curves intersection (PMTP) is observed in the telecommunication range (1550 nm). Calculation by the effective index method.

Based on this expression, we cannot expect a strong increase in the sensor sensitivity based on the intermodal interaction. Consequently, the phenomenon being discussed is precisely related to the presence of waveguide dispersion. To emphasize this fact, it is suggested that the studied phenomena be referred to as the “effect of dispersion-enhanced sensitivity”. This is because the dispersion properties of the fundamental and the first modes of the silicon waveguide are strongly different, and the dependence of their group index (velocity) on the wavelength provides the possibility that the values N_0g_ and N_1g_ can be matched to each other in the telecom wavelength range (see Figure 2). This condition of matching the group indices of the interacting waves is usually called the phase matching turning point (PMTP) [28]. It is characterized by the fact that, in the vicinity of this point, according to Equation (8), the sensitivity of the optical sensor undergoes hyperbolic growth.

**Figure 3:**
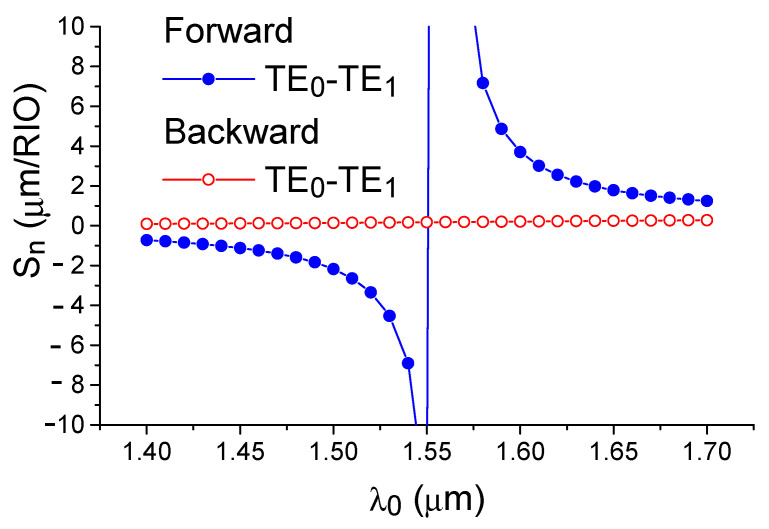
Homogeneous sensitivity dependence on the wavelength. It is seen the resonant behavior of the sensitivity for the forward diffraction, and the monotonic behavior for the backward diffraction. Calculation by the effective index method.

Calculations show that the first waveguide mode is more sensitive to the changes in the refractive index of the environment than the fundamental one. In particular, for a waveguide with the width of 0.55 μm, the following values are typical: ∂N_0_/∂n_c_ = 0.12 and ∂N_1_/∂n_c_= 0.70. For the forward diffraction (TE_0_–TE_1_), the numerator of Equation (8) decreases slightly. At the same time, the denominator of Equation (8) gives it a resonant character and radically increases the sensitivity of the optical sensor for the forward diffraction (see Figure 3). As such, for the backward diffraction process, the sensitivity changes monotonically versus the wavelength and is significantly (by an order of magnitude) lower in magnitude for forward diffraction.

**Figure 4:**
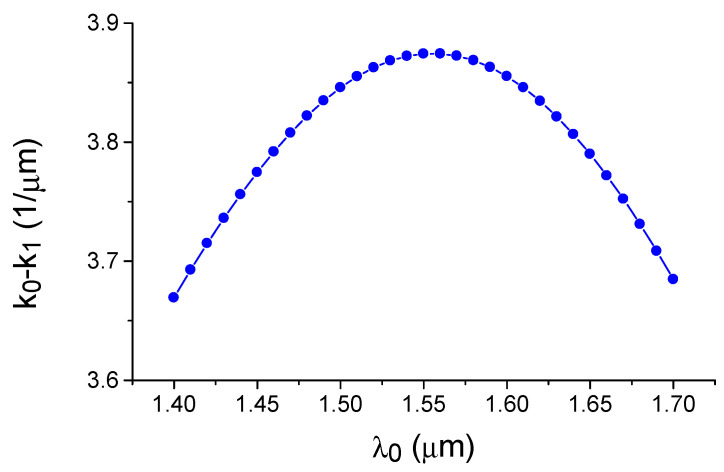
Dependence of the difference between the wave vectors of the fundamental and first TE modes of the silicon waveguide on the wavelength. Calculation by the effective index method.

Physically, the phenomenon of dispersion-enhanced sensor sensitivity can be explained by graphically displaying Equation (1). For this purpose, Figure 4 depicts the dependence on the wavelength for the difference between the wave vectors of the fundamental and the first modes of the silicon waveguide. The condition of phase synchronism corresponds to the crossing of this dependence by the horizontal line corresponding to the diffraction grating wavenumber value (K = 2 π/Λ). Due to the specific dispersion behavior of the mode indices in the silicon waveguide, it can be seen that, for each grating period greater than the minimum value, the phase synchronization condition is observed simultaneously for a pair of optical wavelengths. As the grating period decreases, these wavelengths approach each other in the direction of the extremum where Δk = k_0_ − k_1_ dependence versus the wavelength λ_0_, which corresponds to the PMTP observation condition. The data shown in Figure 2 and Figure 3 were obtained by the effective index method (EIM) [44], which describes the optical properties of three-dimensional strip waveguides in the analytical form with a sufficient accuracy for a qualitative analysis.

Another important optical sensor parameter is the intrinsic limit of detection or iLOD, which is determined by the value of S_n_, as well as the bandwidth δλ. The latter value can be found from the analysis of efficiency η for the collinear Bragg diffraction obtained by the coupled mode method [45].

η = sin^2^[(σL)^2^ + (δβL/2)^2^)^1/2^]/[1 + (k/δ)^2^] (10)

where σ is the coupling coefficient of the diffraction grating and δβ is the mismatch of the wave vectors of interacting waves and the diffraction grating. The bandwidth δλ is usually determined as a change in the wavelength where the efficiency η decreases by a factor of two from the maximum value observed under the condition kL = π/2.

For narrow-band filters, this can be found from the following relationship:

L ∂(k_0_ − k_1_ − K)/∂ω⋅∂ω/∂λ⋅δλ/2 ≈ 1.254 (11)

Based on the determination of the group index and the internal relation ω and λ_0_, we can obtain the desired expression for the bandwidth:

δλ ≈ 0.8 λ_0_^2^/[L⋅(N_0g_ − N_1g_)] (12)

where 0.8 ≈ 2 × 1.254/π. At the same time, the intrinsic limit of detection is found by taking into Equation (8) into account:

iLOD = δλ/S_n_ ≈ 0.8 λ_0_/[L⋅(∂N_0_/∂n_c_ ± ∂N_1_/∂n_c_)] (13)

Note that, despite the resonant behavior of the sensitivity, the expected intrinsic limit of detection still remains finite due to the similar resonant properties of the filter element of an optical sensor, although both of these parameters undergo hyperbolic growth; however, when determining the sensor sensitivity, they completely compensate each other.

## 3. Numerical Modeling

**Figure 5:**
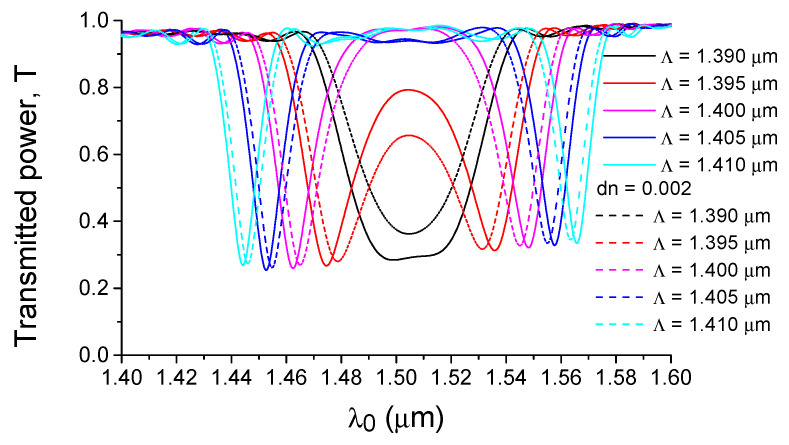
Dependence on the optical wavelength for the transmitted power (T) of the fundamental mode of a silicon waveguide (h = 0.25 µm, w = 0.55 µm) surrounded by water and for water with a small change (dn = 0.002) in the refractive index. Calculation by the 3D FDTD method.

**Figure 6:**
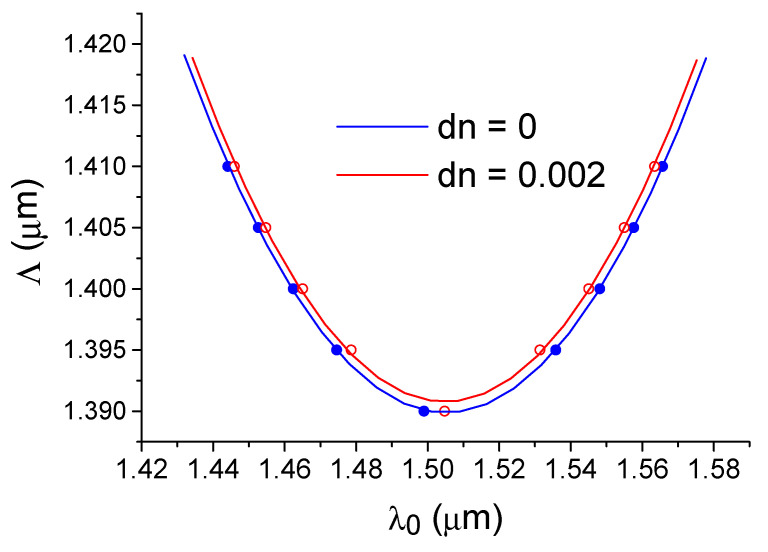
Dependence of the diffraction grating period on the optical wavelength for which Bragg diffraction occurs from the TE_0_ mode to the TE_1_ mode. The structure is surrounded by distilled water and for water with a small change (dn = 0.002) of the refractive index. Calculation by the 3D FDTD method.

The effect of dispersion-enhanced sensor sensitivity is illustrated by direct calculations using 3D FDTD, where the diffraction with a change in the mode number for a silicon waveguide with an asymmetric diffraction grating (see Figure 1b) is presented in Figure 5. It can be seen that when approaching the PMTP condition, not only does the sensitivity of the sensor (wavelength shift with the change in the refractive index of the environment) increase, but also the filter bandwidth (see Figure 5). Physically, this is related to the uncertainty relation for the wave vector (due to the finite length of the diffraction grating), and with the change in the slope of the dispersion dependence of Δk(λ) shown in Figure 4 or the equivalent dependence of Λ = 2 π/Δk(λ) shown in Figure 6. In the PMTP condition, we see a maximum diffraction bandwidth and the limit of endless sensitivity growth, which is formally seen in the Equation (8) for the case when the values of group indexes for both interacting modes are the same.

**Figure 7:**
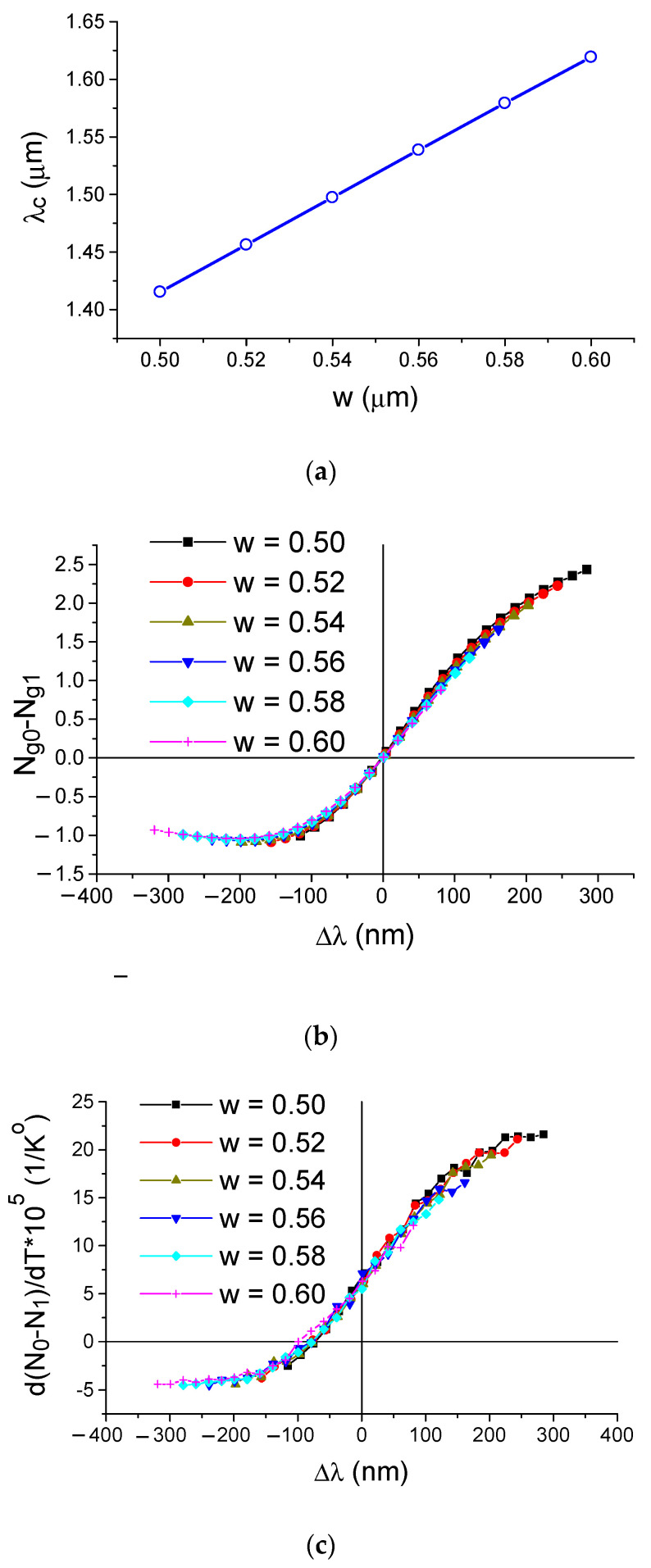
Critical parameters of the bimodal sensor. (**a**) Dependence of λ_c_ on the waveguide width w. (**b**) Dependence of the difference for TE_0_ and TE_1_ group modes index on the cover index. (**c**) Dependence of the effective mode index temperature sensitivity on the optical wavelength increment Δλ = λ – λ_c_. Simulation by 3D FEM.

Let us follow the diffraction peak positions with a change in the grating period and/or refractive index of the water environment (see Figure 6). Near the extremum of dependence Λ(λ), minimal changes in the optical wavelength or refractive index provide a maximum change in the Bragg wavelength, which is a graphical justification for the effect of dispersion-enhanced sensor sensitivity. In addition, from the data shown in Figure 6, it is possible to calculate the homogeneous sensitivity S_n_ (see Figure 7a), which increases in a hyperbolic way as a function of Δλ = λ − λ_c_, where λ_c_ is the wavelength at which the group indices of the fundamental and first modes coincide (PMTP condition). The data of Figure 7a were obtained by the EIM approximation, which helps to quickly analyze sensor structures with a wide variety of parameters. In particular, calculations of structures with different silicon waveguide widths show that the sensitivity of the sensor is mainly determined by the wavelength deviation Δλ from the PMTP point. At the same time, the maximum sensitivity in a real situation will always be limited to the finite bandwidth observed in the diffraction with a change in the mode number (TE_0_–TE_1_).

**Figure 8:**
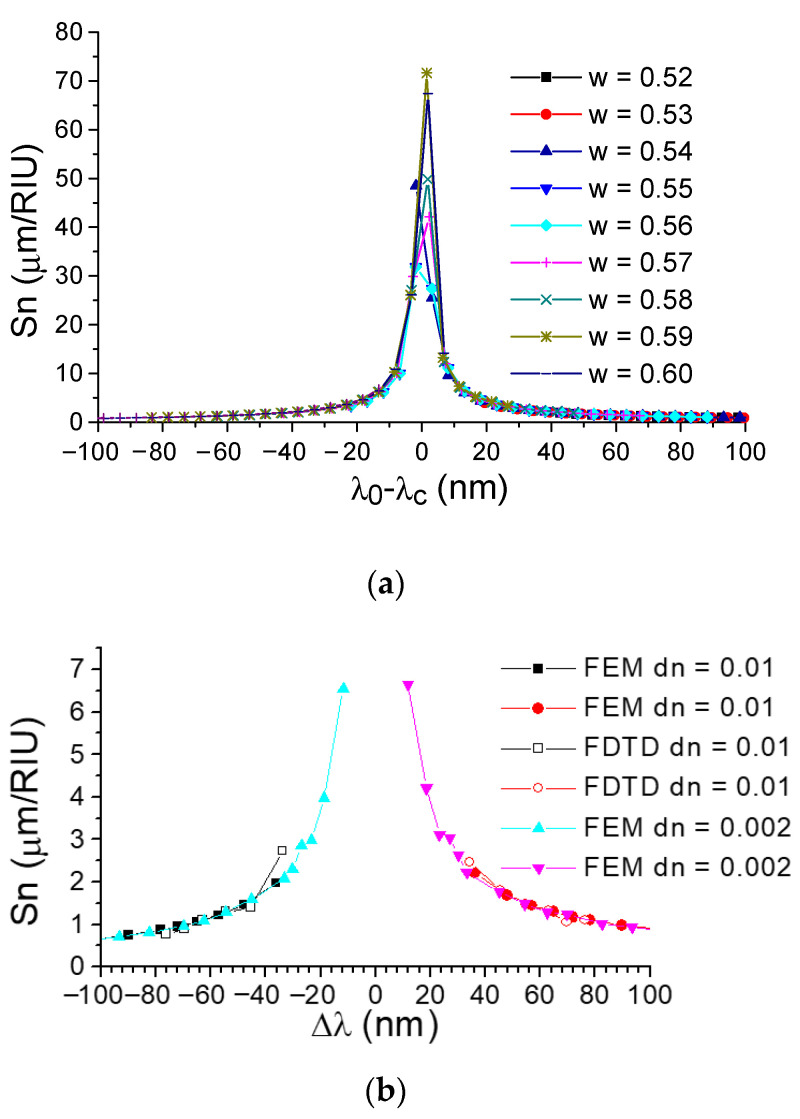
Dependence of the absolute value of the homogeneous sensitivity S_n_ on the optical wavelength increment Δλ = λ − λ_c_ for the collinear Bragg diffraction from the TE_0_ mode to the TE_1_ mode for the structure from Figure 1b when surrounded by distilled water with a small perturbation (dn = 0.002) of its refractive index. (**a**) Calculation under the EIM approximation for infinitive grating. (**b**) Calculation by the 3D FEM method for mode indexes (infinitive grating) and by the 3D FDTD method (compact grating with length of 172 μm) for wavelength responses.

The properties of real three-dimensional waveguide structures were analyzed using numerical three-dimensional algorithms, including the finite difference method (to analyze waveguide mode indices) and the finite difference time domain method (to analyze spectral dependences). The data of these calculations are shown in Figure 7 and Figure 8. The results of the FEM simulations (see Figure 7) describe the general properties of an arbitrary bimodal sensor with an asymmetric grating (see also Equation (8)). The sensitivity is strongly dependent on the shift of Δλ regarding the sensor optical wavelength related to the phase matching turning point λ_c_ as presented in Figure 7a. This determines the difference in the group indexes of TE_0_ and TE_1_ modes, which are mainly dependent on Δλ and have a slow dependence on the waveguide dimensions as shown in Figure 7b. The refractive index of silicon has a high dependence on temperature. Thus, one has to consider the influence of the temperature on the measurement of results. The bimodal sensor provides a self-compensation effect when the temperature dependences of the TE_0_ and TE_1_ modes are summarized with the different signs. The resultant temperature contribution to the sensitivity of bimodal sensor is about 1/8 of the alternative Bragg grating sensor that utilize back diffraction of TE_0_ mode. Nevertheless, in sensor design, one must take into account the systematic error, which can be as much as about 2 × 10^−4^/K° due to the mode index dependence on the temperature. At the same time, it is possible to construct the temperature independent sensor by using the low wavelength shoulder (Δλ = −75 nm) shown in Figure 7c.

The main sensor features were examined by direct 3D FDTD numerical modeling. In particular, the calculation by the 3D FDTD for the short structure with 128 grating grooves and the period of 1.3395 µm shows that the sensitivity reaches 3 µm/RIU (see Figure 8) with a strong tendency to a further increase in the sensitivity for longer diffraction gratings, giving a narrow band filtering and provides the possibility to work at wavelengths close to the PMTP point. For example, we can expect the sensitivity of 6 µm/RIU for Δλ = 12 nm, 26 µm/RIU for Δλ = 3 nm. It is important to understand that a hyperbolic increase in sensitivity will not lead to an increase in the intrinsic limit of detection, which will still be determined by the diffraction grating length and the change in the effective refractive index of interacting modes to the variations in the refractive index of the environment (see Equation (13)).

The values of S_n_ = 3 µm/RIU obtained during the 3D FDTD numerical modeling significantly exceed the capabilities of the best alternative optical sensors based on single-mode slot or SWG waveguides, which also have significantly more stringent requirements for the manufacturing technology due to the need for a high-precision etching of narrow grooves (about 100 nm) in silicon.

## 4. Conclusions

This paper has considered the modern conception that proposes a move away from the usual paradigm of only using single-mode waveguides and has considered a way to dramatically increase the sensitivity of optical sensors based on studying the intermodal interaction of guided optical waves in a two-mode optical waveguide in the presence of an asymmetric periodic perturbation of the refractive index. The design here implements an asymmetric segmented grating that is placed on a two-mode silicon waveguide. Such a system has unique dispersion properties that leads to the implementation of collinear Bragg diffraction with mode number transformation in which there is an extremely high Bragg wavelength dependence on the refractive index change of the environment. This is caused by the presence of a dispersion maximum for the difference between the fundamental and first mode wave vectors of the silicon waveguide as a function of the optical wavelength. If the diffraction grating wave vector is close to the condition of observing this maximum (PMTP), then minimal changes in the properties of the waveguide and/or its environment will give an unusually high change in the Bragg wavelength. This effect of dispersion-enhanced sensitivity is suitable for creating promising types of novel optical sensors. The features of this type of intermodal interaction in silicon waveguides have been studied by numerical methods using the commercial optical package RSoft-SYNOPSYS [43]. Conditions under which high diffraction efficiency are reached with a change in the mode number have been found, and the effect of dispersion-enhanced sensitivity is observed in the telecommunication wavelength range.

It is shown that the effect considered here can be used to create optical sensors with high homogeneous sensitivity (about 3 µm/RIU for a sensor with the sensor element length of 172 μm) with the potential of further sensitivity increases (up to 50 µm/RIU) for longer structures. The proposed sensors present a sensitivity many times higher than that offered by single-mode waveguides and the required hardware can be manufactured by the standard CMOS-compatible technology used in silicon photonics today [1,2,3,4,5,6,45,46,47,48]. At the same time, this technology does not increase the intrinsic limit of detection (iLOD) when compared to alternative types of optical sensors. This sensor design provides a relatively low contribution regarding temperature effects on the measured index (<2 × 10^−4^/K°). Besides, the principal possibility exists to construct a temperature-insensitive bimodal sensor by using the low wavelength peak of the sensor response.

The advantage of such intermodal interaction-based sensors is that, due to their high sensitivity, a high resolution of sensing may be reached with a significantly low spectral resolution of the measuring system, which opens up the possibility for their wide distribution due to the simplification and cheaper interrogation equipment.

## Data Availability

The Photonic Design Software and all data are provided by Rsoft/Synopsys by the single license. Information about the software is available online: https://www.synopsys.com/optical-solutions/rsoft.html.
